# Activated Platelets Express GPI-Anchored Fibrocystin-L (PKHD1L1), from Granules, Absent or Deficient in Paroxysmal Nocturnal Hemoglobinuria

**DOI:** 10.3390/ijms27177796

**Published:** 2026-08-31

**Authors:** Janos Polgar, Jeannine M. Clemetson, Edith Magnenat, Timothy N. Wells, Helena Röss, Sophie Rochat, Lorenzo A. Alberio, Kenneth J. Clemetson

**Affiliations:** 1 Theodor Kocher Institute, University of Berne, Freiestrasse 1, CH-3012 Berne, Switzerland; 2 Serono Pharmaceutical Research Institute, 14, Chemin des Aulx, Plan-les-Ouates, CH-1228 Geneva, Switzerland; 3 Medicines for Malaria Venture, 20 Route des Pre-Bois, CH-1215 Geneva, Switzerland; 4 Topographische und Klinische Anatomie, Institute of Anatomy, University of Berne, CH-3012 Berne, Switzerland; 5 Department of Haematology, Department of Biomedical Research, Inselspital, University of Berne, CH-3008 Berne, Switzerland; 6 Department of Haematology, Centre Hospitalier Universitaire Vaudois CHUV, University of Lausanne, CH-1011 Lausanne, Switzerland

**Keywords:** platelets, granules, receptors, complement, paroxysmal nocturnal hemoglobinuria, fibrocystin L, glycophosphatidylinositol-(GPI-)anchored

## Abstract

Platelets express several glycophosphatidylinositol-(GPI-) anchored receptors, mainly involved in protection against lysis by activated complements. Most of these are well-characterised and are expressed on other blood cells. More recently, CD109 with a mass of 175 kDa was also shown to be a GPI-anchored receptor, surface expressed only on activated platelets, with a role as a co-receptor for transforming growth factor-β (TGF-β). CD109 is expressed on a wide range of cells and is a marker for various types of tumors. Activated platelets express an even larger GPI-anchored receptor at about 500 kDa. We have now isolated this and identified it as fibrocystin L, also known as polycystic kidney hepatic disease L1 (PKHD1L1). Fibrocystin L, like other platelet GPI-anchored receptors, is missing or deficient in paroxysmal nocturnal hemoglobinuria. Fibrocystin L is also expressed in activated T-cells and may be involved in immune responses. Recently, there have been additional reports of PKHD1L1 expression and roles as a coat protein of hair-cell stereocilia essential for normal hearing, as well as reports of them in the dentate gyrus in mice involved in susceptibility to seizure. In all these cases, GPI anchors were not reported, but neither were they tested for. During the fluorescence microscopy studies, we used CD109 as a control and observed that it is also a granule protein that had not been previously reported.

## 1. Introduction

Platelets are protected against lysis by activated complements by expressing several glycophosphatidylinositol-(GPI-)anchored receptors that neutralize complement components. These include CD55 (decay accelerating factor, DAF [1] (70 kDa), CD59 [2] (20 kDa), and C8-binding protein [3] (65 kDa)). More recently, CD109 (175 kDa) was described as a GPI-anchored receptor [4] expressed on activated platelets with a role as a co-receptor for TGF-β [5]. Tissue factor pathway inhibitor (TFPI) is also expressed on activated platelets [6], but indirectly via binding to a GPI-anchored protein [7]. Semaphorin 7A has been reported to be expressed on platelets in α-granules and shown to have a GPI anchor [8]. Glypicans also have GPI anchors, but their expression on platelets is less well studied. TFPI prevents thrombin generation by blocking the active site of FVa and is modulated by protein S [9,10,11,12,13]. There are two splice variants: the TFPIβ and TFPIα. All these proteins are absent or deficient in paroxysmal nocturnal hemoglobinuria (PNH), a clonal disorder in which the gene for the synthesis of the GPI anchor is mutated and which, when untreated, has a poor prognosis [14]. GPI-anchored receptor expression is affected on a range of blood cells in PNH, including erythrocytes, leukocytes, and platelets. The effects of lack of GPI-anchored receptors on platelets are poorly understood [15]. It is likely to lead to platelet lysis by activated complement and could therefore be a major reason for the thrombosis often associated with this disorder [16]. Lysis of erythrocytes releases large amounts of ADP, a major platelet agonist. Normally, small amounts of ADP coming from erythrocytes or activated platelets are quickly removed by apyrase activity (CD39) on the endothelial surface [17]. In PNH, this may be overwhelmed, leading to massive platelet activation and to thrombus formation. Platelets also express CD46 (transmembrane membrane cofactor protein), providing some residual protection against complements in PNH [18].

Life-threatening PNH is often treated with eculizumab, a monoclonal antibody directed at the activated complement, which thus prevents blood cell lysis [19]. Despite this prevention of lysis, there are reports of thrombotic events in PNH patients, leading to suggestions that the absence of other GPI-linked molecules, which normally have an anti-thrombotic role, might be responsible. Clearly, TFPI would be a candidate for this role. Whether platelets express tissue factors remains a highly controversial area, but, even if they do not, the absence or decrease of TFPI expression in PNH would be expected to shift towards a more thrombotic phenotype.

During earlier studies with PNH platelets, we identified a high-molecular mass glycoprotein, GP500, that was also absent in this disorder and therefore likely to be GPI-anchored [20]. It could also be removed from normal platelets using glycosylphosphatidylinositol-specific cleavage. We describe here the isolation of this glycoprotein from platelets using affinity chromatography and gel filtration and its identification as fibrocystin L by protease digestion, fractionation of peptides by high-performance liquid chromatography (HPLC), and sequencing of major peptide fractions. Expression in resting and activated platelets was investigated by flow cytometry using a polyclonal rabbit antibody to the isolated fibrocystin L glycoprotein. Location in resting platelets was investigated by fluorescence confocal microscopy.

Fibrocystin, encoded by polycystic kidney and hepatic disease gene 1 (PKHD1), is a large (4074 aa), putative receptor molecule involved in autosomal-recessive polycystic kidney disease [21]. Typical symptoms in neonates are very much enlarged kidneys due to dilation of collecting ducts, but the function of the protein remains unknown. Fibrocystin L is the only known homolog and is also predicted to be a very large receptor protein (4243 aa), with 25% identity and 41.5% similarity [22]. The only well-characterized domain in the fibrocystins is the immunoglobulin-like fold shared by plexins and transcription factors (TIG/IPT), which is also found in some structurally related molecules. Expression analysis showed that fibrocystin L is expressed in activated, but not in resting, T-lymphocytes [22]. PKHD1L1 is expressed as a coat protein of hair-cell stereocilia that is essential for normal hearing [23] and in the dentate gyrus in mice where it is involved in seizure susceptibility [24].

## 2. Results and Discussion

**Figure 1:**
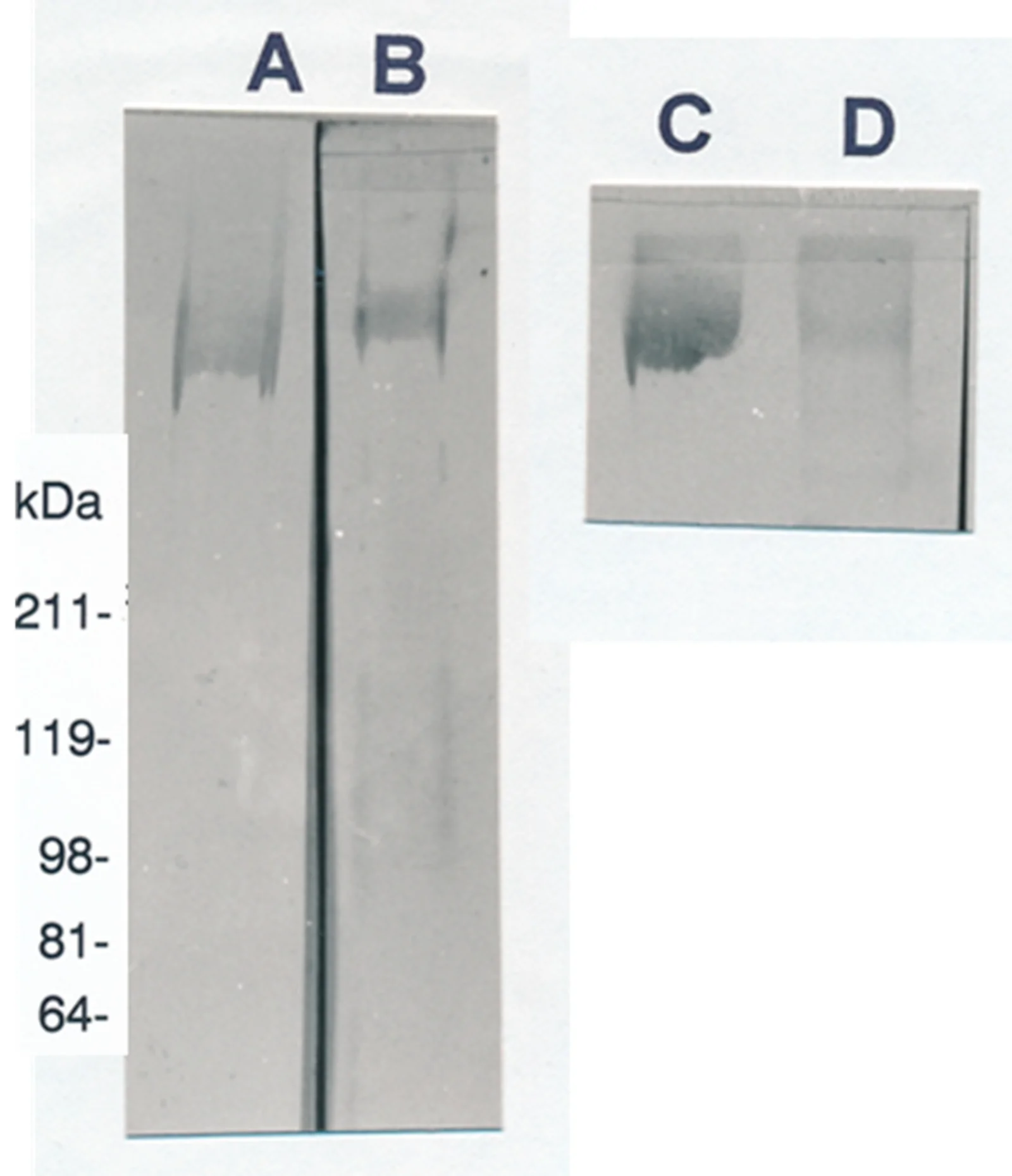
Detection of fibrocystin L by SDS-PAGE/western blotting with rabbit anti-fibrocystin L polyclonal antibodies. A,B: Platelet lysate (10^8^ platelets). C: Supernatant of PI-PLC-treated platelets (5 × 10^8^ platelets). D: Platelet pellet from PI-PLC-treated platelets (5 × 10^8^ platelets). SDS-PAGE was under non-reducing (A) or reducing conditions (B–D). Note that the fibrocystin L detected in the reduced sample (B) had a higher apparent molecular mass than that in the non-reduced sample (A). This is likely due to the presence of large disulfide-linked loops in fibrocystin L. There are no appropriate pre-stained molecular mass markers in the fibrocystin L size range.

In these studies, we demonstrate that the 500 kDa GPI-linked glycoprotein was first detected by its absence/deficiency in platelets from PNH patients, fibrocystin-L is the product of the PKHD1L1 gene, and that, in platelets at least, it is GPI-anchored. In Figure 1, the fibrocystin-L detected in the reduced sample (B) had a higher apparent molecular mass than that in the non-reduced sample (A). This is likely due to the presence of large disulfide-linked loops in fibrocystin-L. The molecular mass is an estimate because there are no appropriately sized pre-stained molecular mass markers in the fibrocystin-L size range. The fact that fibrocystin-L is so large may account for the fact that it was not detected earlier on gel electrophoresis of platelets, as it did not enter the gels used.

**Figure 2:**
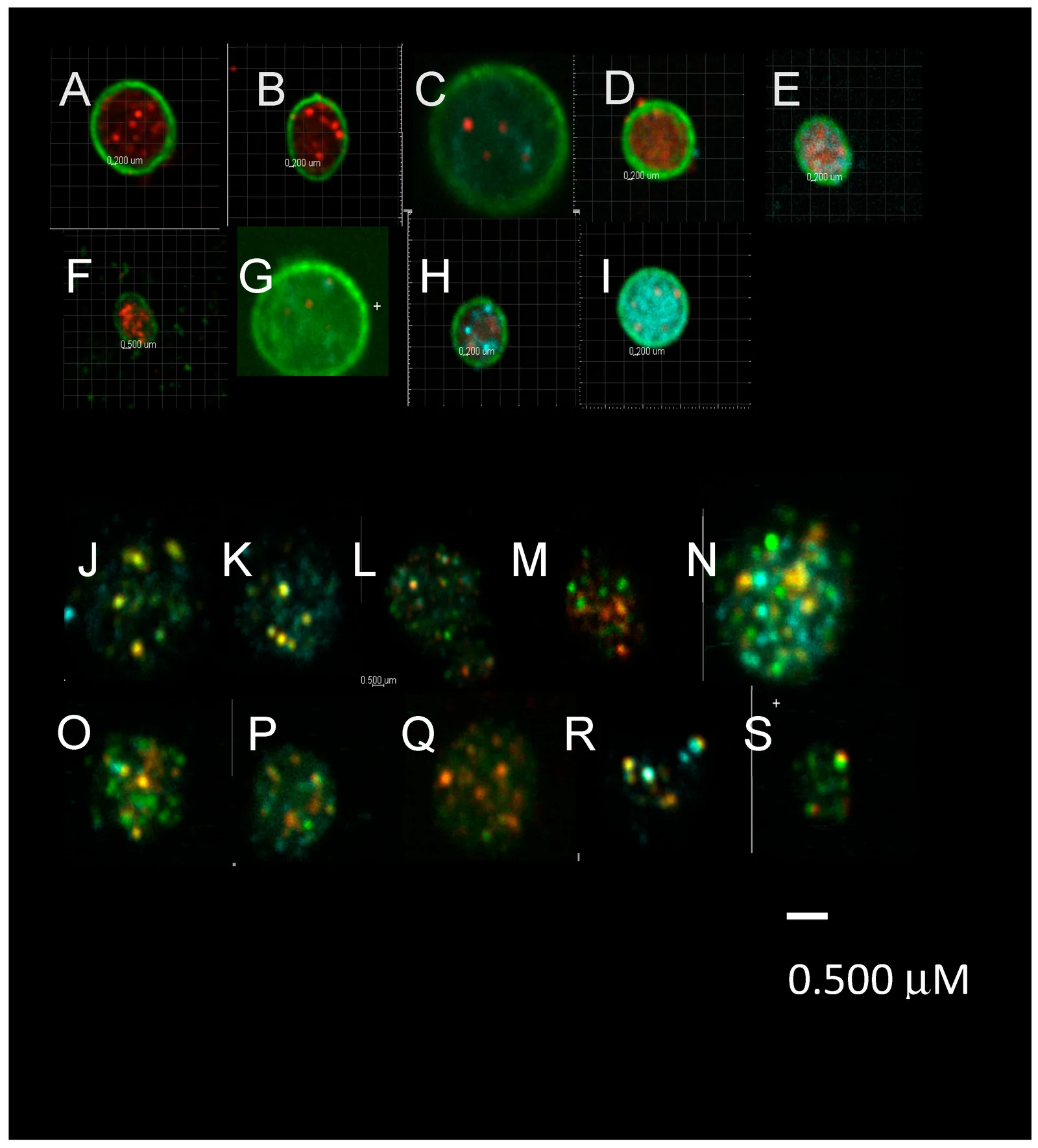
Top Panel (**A**–**I**) Confocal microscopy of fixed, permeabilized, resting platelets stained with Abcam goat anti-β-tubulin antibodies and Abcam donkey anti-goat Alexa 488-labelled secondary antibodies. (**A**–**D**) In addition, it was stained with rabbit anti-fibrocystin L antibodies and mouse anti-rabbit STAR 635-labelled second antibodies. (**D**–**F**) was further stained with mouse anti-VWF antibodies and goat anti-mouse STAR Red 580-labelled secondary antibodies. The green-stained tubulin delineates the platelet margin, whereas the orange red VWF/red fibrocystin L stain is located in the platelet granules. (**G**–**I**) In addition, anti-β-tubulin was also stained (**G**) with mouse anti-P-selectin antibodies and anti-mouse Alexa 488-labelled secondary antibodies and (**H**) stained with mouse anti-CD109 antibodies and anti-mouse Alexa 405-labelled secondary antibodies (**I**) stained with rabbit anti-fibrocystin L antibodies and mouse anti-rabbit STAR 635-labelled second antibodies and mouse anti fibrinogen antibodies and anti-mouse Alexa 405-labelled secondary antibodies. Bottom Panel (**J**–**S**) Fluorescence microscopy of fixed, permeabilised, resting platelets (**J**–**L**) stained with rabbit anti-fibrocystin L antibodies and mouse anti-rabbit Alexa 568-labelled secondary antibodies and with mouse anti-CD109 antibodies and anti-mouse Alexa 488-labelled secondary antibodies (**M**,**N**) stained with rabbit anti-fibrocystin L antibodies and mouse anti-rabbit Alexa 488-labelled secondary antibodies further stained with mouse anti-VWF antibodies and goat anti-mouse STAR Red 580-labelled secondary antibodies. (**O**,**P**) Stained with rabbit anti-fibrocystin L antibodies and mouse anti-rabbit Alexa 488-labelled secondary antibodies and with mouse anti VWF antibodiand goat anti-mouse STAR Red 580-labelled secondary antibodies, (**Q**) stained with rabbit anti-fibrocystin L antibodies and mouse anti-rabbit Alexa 488-labelled secondary antibodies and with mouse anti-CD109 antibodies and anti-mouse Alexa 568-labelled secondary antibodies. (**R**,**S**) Stained with mouse anti-CD109 antibodies and anti-mouse Alexa 488-labelled secondary antibodies. Controls with secondary antibodies alone were essentially negative (general faint diffuse stain not concentrated in granules).

In addition, we show by flow cytometry that fibrocystin L is only expressed when platelets are activated. Using fluorescence confocal microscopy, we show that the rabbit anti-fibrocystin polyclonal antibodies specifically label spherical regions within the platelets that correspond to granules—most likely α-granules. At present, very little is known about fibrocystin L function, either in platelets or in T-lymphocytes where it was originally detected after activation [22]. Nor is it clear whether the T-lymphocyte fibrocystin-L is GPI-anchored, although this is probably the case. GPI-anchored receptors are known to localize to lipid raft domains in the membrane and to signal via these to the cell interior. Over 170 GPI-anchored mammalian proteins are known, and fibrocystin L would appear to be the largest yet detected [25]. CD109 is also a GPI-linked receptor, and we used it as a control in our fluorescence microscopy experiments as shown in Figure 2. These results indicate that, like semaphorin 7A and fibrocystin-L, CD109 is also an α-granule membrane receptor. This would also be the first time this has been shown. The presence of fibrocystin L in both platelets and T-lymphocytes may indicate a role in hemostasis/thrombosis or in immunology/inflammation. As with many platelet proteins lacking a clearly established function yet being well-preserved during evolution, a role in defense against parasites may be suspected but difficult to establish.

The expression of PKHD1L1 as a coat protein of hair-cell stereocilia that is essential for normal hearing [23] and in the dentate gyrus in mice where it is involved in susceptibility to seizure [26] was also not investigated for the presence of a GPI anchor. In the case of platelets, the absence of PKHD1L1 (fibrocystin-L) in PNH was important in indicating that a GPI anchor was present and needed further investigation. GPI anchors may be present in the other cases of PKHD1L1 expression but will require testing.

The physiological role of fibrocystin-L is a major challenge. It was first detected as an inducible protein in lymphocytes as a homolog of fibrocystin (PKHD1), and its sequence was made available in data banks. One of the first consequences of this was that the sequence indicated that PKHD1 was a large (4072 amino acids) membrane-bound molecule with a single transmembrane domain and a 140 amino acid cytoplasmic domain. Several IPT domains are present. They consist of an immunoglobulin-like fold, and proteins that contain IPT domains generally belong to one of two classes: intracellular DNA transcription factors (the Rel family) or single-pass cell-surface receptors members of the large family of plexins [27]. The molecular structure and function of fibrocystin is extensively discussed in Bannell and Cockburn [28] and in Gulati et al. [29]. We also examined the sequence using the Fankhauser and Maeser program [30], but in contrast to PKHD1L1, it was strongly predicted that it does not have a GPI anchor. While most recent studies on PKHD1 have concentrated on its role in autosomal recessive polycystic kidney disease, PKHD1L1 has been investigated as a human deafness gene. PKHD1L1 is a stereocilia protein necessary for the transient stereocilia surface coat [31]. This study also involved DeepFold2 modelling of a fragment of about a third of PKHD1L1, comprising a TEMED-like domain and a MAD-like domain situated towards the N-terminus. In platelets, PKHD1L1 is evidently present on the surface after activation, which is in agreement with our flow cytometry studies because a study of the platelet sheddome [8] showed it was one of the major proteins released, and inhibiting ADAM17 prevented release. This implies that soluble PKHD1L1 is released to the circulation after platelet activation during hemostasis or vascular disease and may interact with the endothelium or other cells in the circulation to modify their behavior.

A divergent SEA (sea urchin sperm domain) group was found in proteins, including human fibrocystin and fibrocystin-L protein. Fibrocystin and fibrocystin-L possess several TIG domains, two G8 domains, and two regions of β-helix repeats. The SEA domain in human fibrocystin-L lies in the C-terminal part of the extracellular region before an Ig-like domain. Like some of the Notch receptors, both human fibrocystin and fibrocystin-like proteins have the Rx[KR]R motif in the loop region between the second and third core β-strands of the predicted ferredoxin-like fold. Fibrocystin undergoes Notch-like sequential proteolysis events, including processing by a probable proprotein convertase at the furin-cleavage site, an ADAM metalloproteinase, and γ-secretase. Fibrocystin-L proteins were found beyond Holozoa in several eukaryotic lineages, including green algae, Alveolata, Euglenozoa, and Haptophyta. SEA domains are also present in these proteins, suggesting that the SEA domains in fibrocystin-L proteins have a deep eukaryotic origin [32].

A recent study found heterogenicity in pulmonary lymphatic endothelial cells, with some defined by PKHD1L1 expression and attention, was drawn to a possible role of cilia within the lymphatic drainage system [33]. Thus, in platelets, PKHD1L1 might have a role in clearance involving cilia within capillaries. At the moment, this is quite speculative.

Many recent papers have reported that specific tumors are depleted in a number of genes, and PKHD1L1 is apparently often one of these [34,35]. Thus, PKHD1L1 (among some other genes) could have a protective effect. How is not clear, but it was observed in several different tumors. This stands in contrast to CD109, which is enhanced in a number of tumors [36].

At present, the role of PKHD1L1, not only in platelets but in a number of cells, including lymphocytes, remains obscure. Perhaps cilia play a wider role in vasculature than previously suspected. Even if PKHD1L1 is knocked out in the haemopoietic line of a mouse stain, it will require an appropriate assay to determine fibrocystin-L function in platelets, and at present, this is not obvious.

While recent studies have advanced the mechanisms of α-granules acquiring cargo [37], the biosynthesis of α-granule membranes in megakaryocyte remains obscure [38].

## 3. Methods and Materials

### 3.1. Patients—Ethics Committee Waiver

This study involved the analysis of platelets from three patients diagnosed with Paroxysmal Nocturnal Hemoglobinuria and was carried out in 1993 before the 2011 Swiss Federal on Research involving Human Subjects came into effect and therefore did not require an Ethics Committee approval. The study was carried out with the ethical principle of the Helsinki Declaration and its subsequent amendments concerning informed consent. Subsequent work involved platelets obtained from buffy coats (anonymous donors) purchased from the Red Cross Blood Transfusion service branch in Bern.

### 3.2. Materials

Chemicals, prestained molecular weight standards, and enzymes were from Sigma (St. Louis, MO, USA); purified phosphatidylinositol-specific phospholipase C (PI-PLC) was from Boehringer (Mannheim, Germany), while N-acetylglucosamine and N-ethylmaleimide were from Fluka, Buchs, Switzerland.

### 3.3. Platelets

Platelets from healthy volunteers were isolated from buffy coats less than 12 h after blood collection, obtained from the Swiss Red Cross Blood Transfusion Service, prepared from citrate-anticoagulated blood. Platelets were purified by differential centrifugation. For resting platelets, PGE1 (1 µM) and/or apyrase (0.2 U/mL of final concentration) were added. These were omitted for platelet preparations that were to be activated. Platelets from four patients (female, between 23 and 34 years old) diagnosed as having paroxysmal nocturnal hemoglobinuria (PNH) by clinical criteria with current platelet counts of 11, 24, 140, and 167 × 10^9^ platelets/L and from controls were isolated from EDTA-anticoagulated blood samples less than 4 h after blood collection. Platelet-rich plasma was used for flow cytometry and inhibitors omitted. Platelet purity was assessed in earlier studies using a microscope and a hemocytometer, and later using an electronic particle counter. Contamination with leukocytes was generally lower than 1 leukocyte per 10^6^–10^7^ platelets. Different control platelets were used for each patient’s platelets in labelling and electrophoretic analysis.

Supernatants were analyzed by SDS-PAGE/silver staining or by SDS-PAGE/fluorography. For some experiments, platelets were activated with thrombin receptor-1 (PAR-1) peptide SFLLRN (10 μM) at 37 °C for 5 min.

### 3.4. Antibodies

Polyclonal antibodies to fibrocystin L were prepared by injection of 20 μg of the isolated molecule, first in the presence of adjuvant, and then in its absence, at weekly intervals for 4 weeks, into the rear foot pad of two rabbits. After checking specific antibody formation by Western blotting against whole platelet lysate, the rabbits were bled every 2 weeks with re-immunization, if required. IgG was isolated from serum by ammonium sulfate precipitation, followed by dialysis.

Fluorescence-labelled antibodies were from Abcam (Cambridge, UK) and Abberior (Goettigen, Germany).

Flow cytometric analysis was performed on a BD FacsCanto flow cytometer (Becton Dickinson, Basel, Switzerland) with two lasers: Argon and HeNe, which produce light beams at, respectively, 488 nm and 633 nm. Physical properties (size, granularity) and fluorescence of FITC-marked cells were measured with this configuration. Platelets were identified and gated on a bi-logarithmic scatterplot based upon their forward and side scatters properties and in additional experiments by detection of fibrocystin L, CD109, or CD62P. The fluorescence emission of tagged platelets was evaluated on a single logarithmic histogram. Binding of anti-platelet specific antibodies was assessed with resting and activated (SFLLRN peptide, 10 μM, 5 min) platelets.

### 3.5. Isolation of Fibrocystin-L

**Figure 3:**
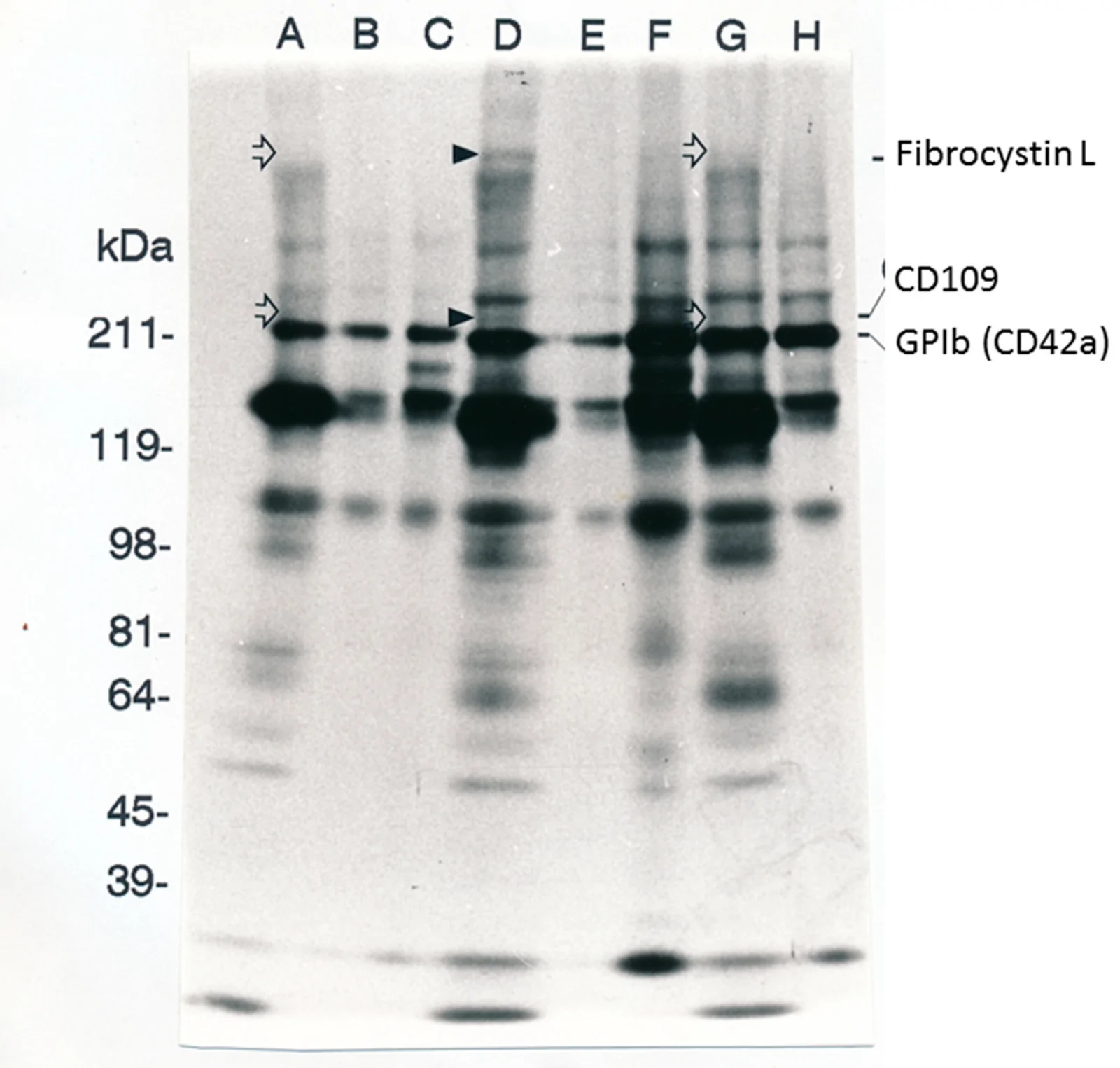
Fluorogram of surface-labelled control and PNH platelet lysates, and supernatants and pellets from surface-labelled control and PNH platelets. All samples were reduced. A–C: PNH patient, A: PI-PLC supernatant, B: Pellet after PI-PLC treatment: C: Platelet lysate, D–H: Control D: PI-PLC supernatant, E: Pellet after PI-PLC treatment: F: Platelet lysate, G,H: Supernatant and pellet from untreated platelets. Samples from a patient and a healthy volunteer were run in 4.5–15% gradient polyacrylamide gel, and the gel was prepared for fluorography. Arrowheads indicate the position of fibrocystin L and CD109. The closed arrowheads indicate presence, and the open arrowheads indicate absence.

**Figure 4:**
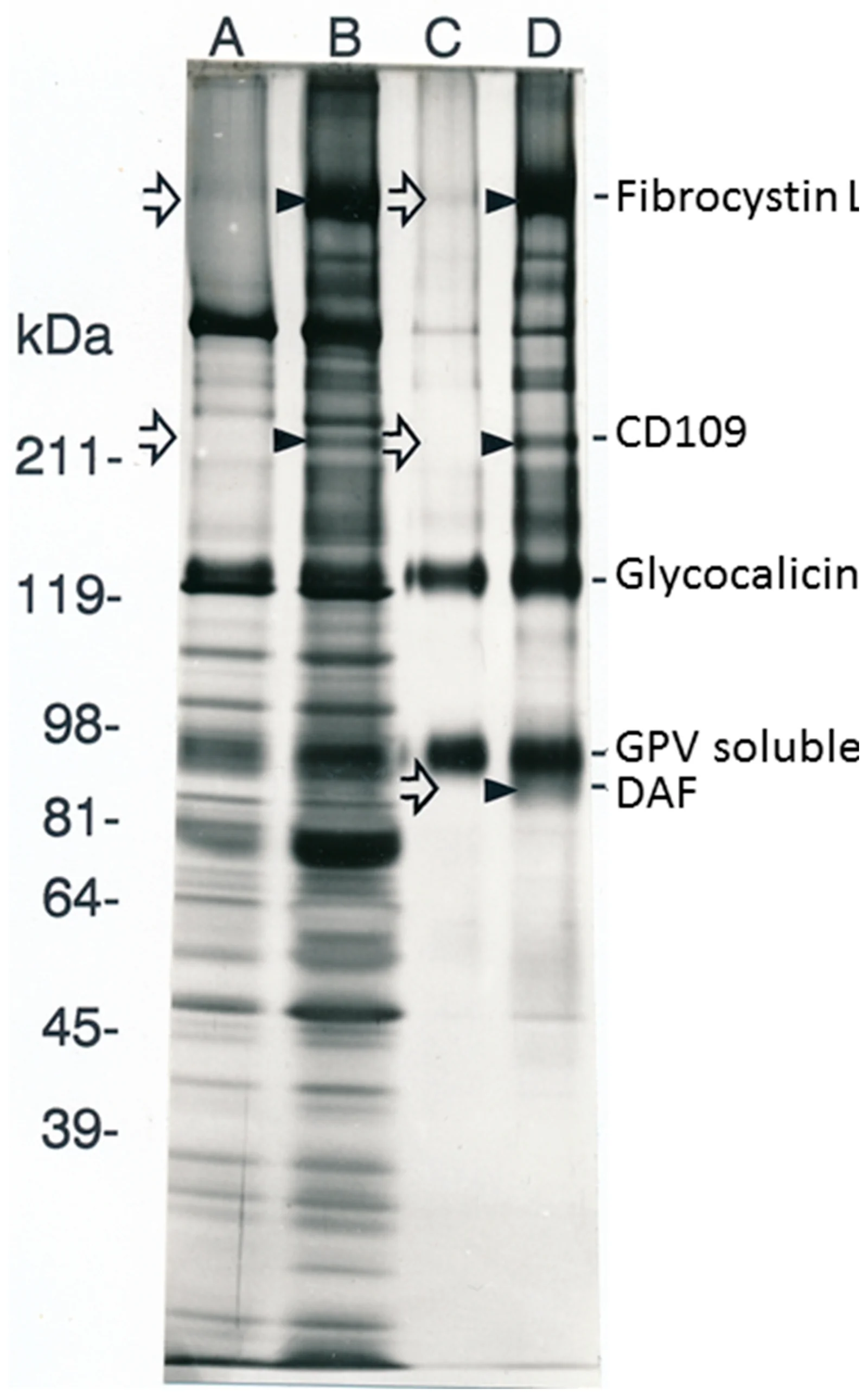
Platelet supernatants and eluates from a wheat germ agglutinin (WGA) column. A: Supernatant of platelets incubated without PI-PLC, B: Supernatant of platelets incubated with PI-PLC, A and B were loaded separately on a WGA column, and the bound material was separately eluted with 2% N-acetyl glucosamine. C: The eluate from WGA using A, D: The eluate from WGA using B: The samples were denatured under reducing conditions and separated on a 4.5–15% gradient polyacrylamide gel that was then silver-stained. The fact that the pre-stained molecular mass markers are not glycosylated, whereas both CD109 and fibrocystin L are, can explain their apparent discrepancies in apparent molecular mass. Arrowheads indicate the position of fibrocystin L, CD109, and DAF. The closed arrowheads indicate presence and the open arrowheads indicate absence.

**Figure 5:**
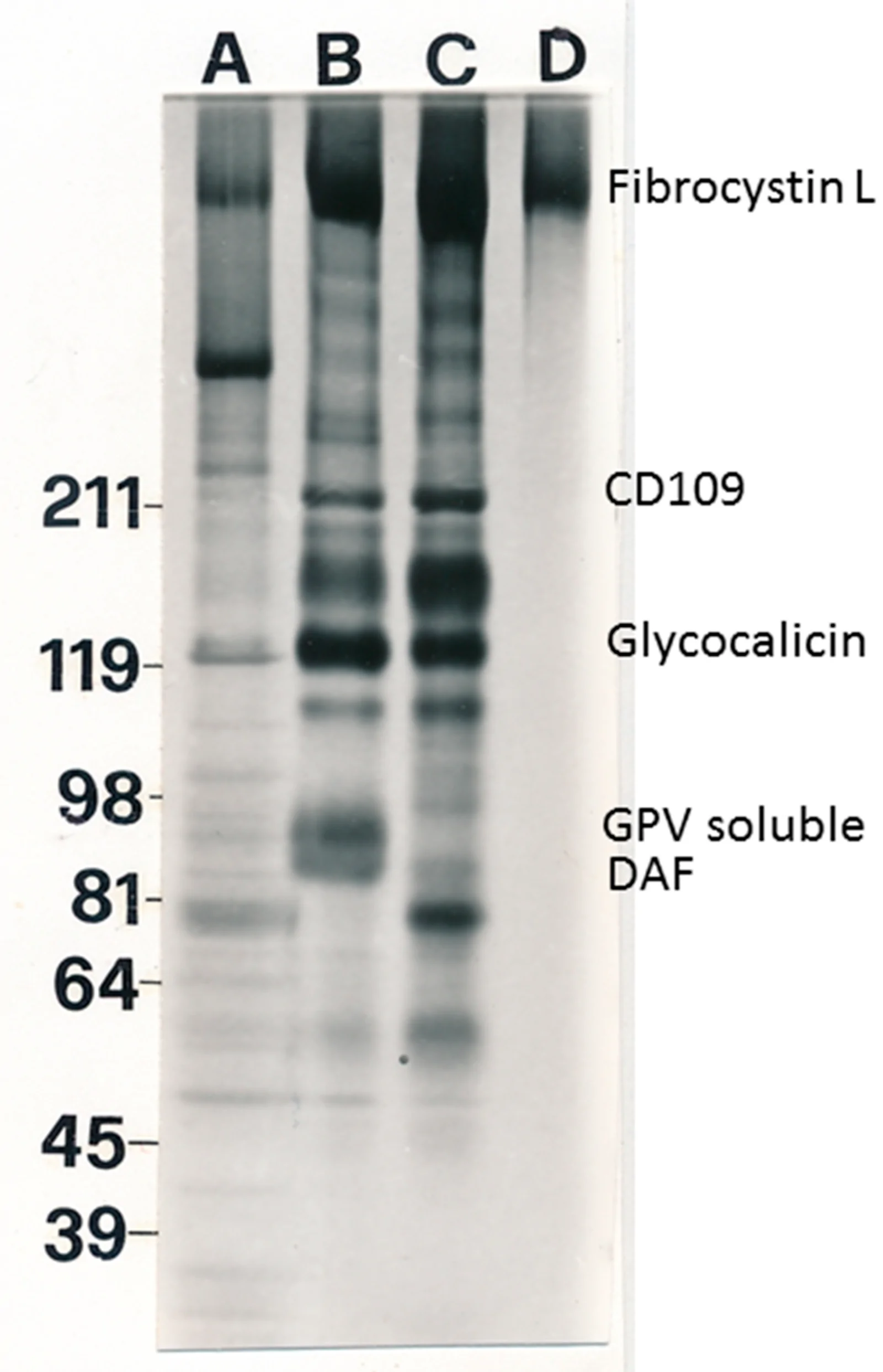
Purification of fibrocystin L from platelets. Samples representing various stages of the purification process were denatured under reducing conditions and separated on a 4.5–15% gradient polyacrylamide gel, which was subsequently silver-stained. A: Supernatant of PI-PLC-treated platelets. B: N-acetyl glucosamine eluate from WGA-affinity chromatography. C: Eluate from DEAE-Sepharose column. D: Eluate of pure fibrocystin L after preparative gel electrophoresis with FMC Prosieve, 4% agarose. The positions of glycocalicin and the soluble fragment of GPV that binds to wheat germ agglutinin were established in earlier work [24].

GP500 (Fibrocystin L) and GP175 (CD109) were both detected as absent or strongly depleted in platelets from paroxysmal nocturnal hemoglobinuria patients (Figure 3) during earlier studies [20]. Based upon these findings, a purification scheme for GP500 could be established (Figure 4 and Figure 5). Platelets were isolated from 20 buffy coats by differential centrifugation in the presence of apyrase to prevent activation by ADP. The isolated washed platelet pellet was treated with 0.2 U/mL of PI-PLC in the presence of 2 mM of N-ethylmaleimide in 4 mM of glucose,120 mM of NaCl, 5 mM of EDTA, and 10 mM of Tris-HCl buffer at 37 °C for 30 min., then solubilized in 25 mL of 2% Triton X-114 in TENA (Tris EDTA NaCl), 2 mM of PMST, and 2 mM of NEM buffer, and the hydrophobic and hydrophilic proteins were separated by TX-114 phase partition [20,39]. Briefly, following solubilization on ice, the platelet lysate was centrifuged (20,000× *g*, 10 min) to remove insoluble material such as cytoskeleton. The supernatant was then warmed to 37 °C for 10 min, and the insoluble material (detergent phase) was removed by centrifugation for 10 min at 20,000× *g*. CD109 was in the detergent phase, and the fibrocystin-L was in the water phase. The supernatant was now separated by affinity chromatography on a wheat germ agglutinin-Sepharose 4B column (2.5 × 20 cm). The column was washed with 0.1% n-nonanyl methyl glucoside (NNMG) detergent (TENA buffer) and then eluted with 2% N-acetyl glucosamine in 0.1% NNMG detergent (TENA buffer). The eluate was concentrated to 10 mL and then separated by ion-exchange chromatography on a DEAE column with an NaCl gradient. The fibrocystin L fractions were concentrated to 1 mL, dialyzed, and further fractionated by preparative gel electrophoresis using a 4% FMC Prosieve agarose gel. The fibrocystin L band was excised and eluted/concentrated by electroelution (Figure 5). The large size of this molecule and its heavy glycosylation precluded many established separation techniques that we tried before.

### 3.6. Identification as Fibrocystin L

The purified GP500 was treated with proteases, and the peptides were separated by HPLC on a C-8 column. The best results were obtained with Lys-C, and the peptides were isolated and sequenced. In this way, these sequences were obtained: (K)STFG(T)FSQFT; (K)DLRQPMVYF(T); (K)GVRTP(G)I; (K)(T)VFLPAD(Y)X(E); where the initial (K) was assumed based on the use of Lys-C. The other amino acids in brackets were uncertain. When the peptide sequences were first obtained (1996), there was no corresponding sequence present in the major sequence data banks. Searches of the data banks were made in subsequent years, and eventually the peptides were identified with the sequences: KSTSGSFSYQFT; KDLSQSMTPFTYA: KSFRTPTIRS; KTVFLPADASE, corresponding to a protein called fibrocystin L, which itself was predicted to be the product of the PKHD1L1 gene present in the 8q23 chromosome region [22] (4243 aa; 466 kDa). If glycosylation mass is also considered, this fits well with the ca. 500 kDa that we originally estimated.

### 3.7. Examination of Fibrocystin L Expression on Platelets Using Rabbit Polyclonal Antibodies

Following the prediction of fibrocystin L protein, several commercial antibodies became available. We tested these in western blotting assays, and, unfortunately, they were (at least at that time) all negative. These antibodies were prepared against a peptide corresponding to the predicted C-terminal sequence. Since it was not known then that fibrocystin L is a GPI-linked protein (at least in platelets), and that this coupling involves cleavage of the C-terminal sequence, this may provide a rational explanation for the poor performance of these antibodies. Recently, a monoclonal antibody against a longer C-terminal sequence was shown to recognize PKHD1L1 [22,23]. Rabbit polyclonal antibodies, prepared against the whole fibrocystin L molecule and isolated from platelets, recognized the appropriate 500 kDa molecule on western blots of whole platelet lysates, and previous treatment of the lysate with phosphatidylinositol-specific phospholipase C (PI-PLC) caused loss or major depletion of this signal in the platelet pellet (Figure 1).

### 3.8. Flow Cytometry Studies on Platelets with Rabbit Anti-Fibrocystin L Antibodies

**Figure 6:**
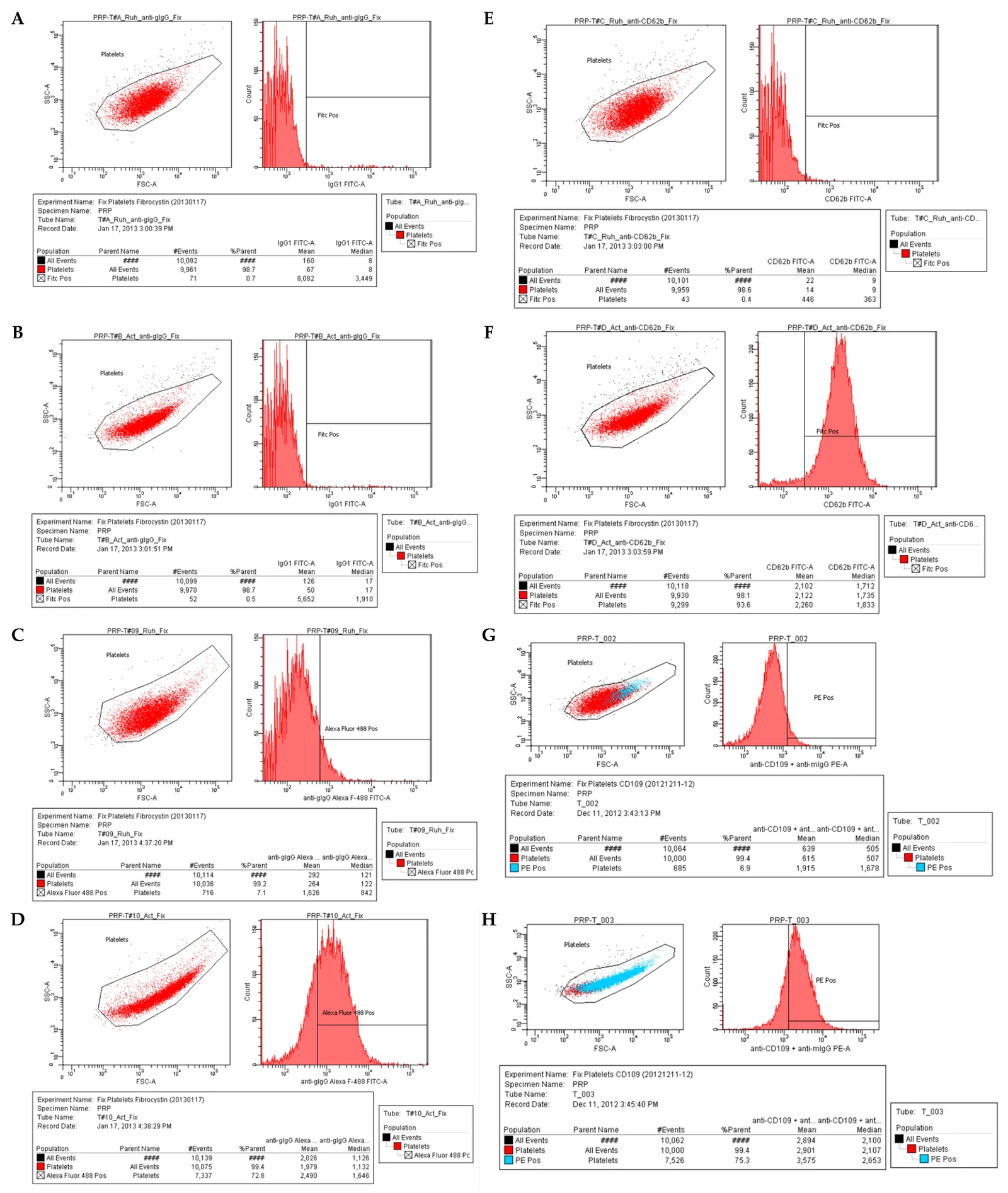
Flow cytometry of resting, fixed platelets (**A**,**C**,**E**,**G**) and activated, fixed platelets (**B**,**D**,**F**,**H**) detected with rabbit IgG and anti-rabbit IgG labelled with Alexa F-488 FITC as second antibody (**A**,**B**). (**C**,**D**); detected with rabbit anti-fibrocystin L polyclonal antibodies and anti-rabbit IgG labelled with Alexa F-488 FITC. The anti-fibrocystin L rabbit IgG was not completely free from dimers, which accounts for the presence of some higher-side scattering (above the main platelet band), as well as the slight shift to higher fluorescence in the resting platelets. (**E**,**F**); detected with mouse anti-CD62P monoclonal antibody and polyclonal anti-mouse IgG labelled with Alexa F-488 FITC. (**G**,**H**): detected with mouse anti-CD109 monoclonal antibody and polyclonal goat anti-mouse IgG labelled with phycoerythrin.

Although we had originally thought that fibrocystin L was expressed on the plasma membrane in resting platelets, further studies indicated that these platelets had been most likely activated during the preparation of washed platelets, and that this required more detailed investigation under more stringent conditions. This was carried out by flow cytometry using the rabbit polyclonal anti-fibrocystin L antibodies (Figure 6).

### 3.9. Proteolysis, Peptide Separation, and Sequencing

Reduced and alkylated protein was digested with Asp-N or Lys-C proteases (Roche Diagnostics, Basle, Switzerland). Urea (3 M final concentration) was added to the mixture to ensure solubility. Peptides from cleavages were separated by reverse-phase chromatography on a C8 column eluted with a linear gradient of 10–70% acetonitrile in 60 min at 1 mL/min. Reduced and alkylated protein was digested with Lys-C or Asp-N proteases, as above. The cleavage mixture was diluted with 10 volumes of water and concentrated in a Speed Vac. The peptides were purified as above.

### 3.10. Sequencing and Mass Spectral Analysis of GP500

N-terminal sequences of peptides from enzymatic and chemical digestions were obtained by Edman degradation with an Applied Biosystem 476A liquid-phase sequencer equipped with on-line phenylthiohydantoin reverse HPLC using an RP 18 column. The molecular masses of peptides were determined by electrospray mass spectrometry on a Trio2/3000 ESI instrument (Thermo Fisher Scientific, Waltham, MA, USA).

### 3.11. Western Blotting Analysis

Cells were lysed in 100 µL of Laemmli buffer. Protein concentrations were quantified by the bicinchoninic acid assay (Sigma) with bovine albumin as a standard. Then, 30 µg of whole cell lysates or nuclear extracts were subjected to SDS-PAGE and transferred to polyvinylidene difluoride membrane (Immobilon-P, MilliporeSigma, Darmstadt, Germany). The immuno-reactive proteins were detected by the enhanced chemiluminescence detection system (ECL, Pierce, Rockford, IL, USA).

### 3.12. Platelet Staining for Fluorescence Microscopy

Cover slips were coated with 50 μL of 0.1% polylysine solution, plus a small amount of detergent to aid spreading to cover whole area. They were allowed to dry, then 30–50 μL of formaldehyde-fixed platelet suspension (ca. 5 × 10^7^/mL) were spread on each polylysine-treated cover slip and allowed to dry ca. 1 h at room temperature. Cover slips were washed overnight in PBS in 50 mL Falcon tubes. Resting platelets were permeabilized with 0.1% Triton X-100 in PBS for 2 min. After each stage, the cover slips were washed in 50 mL Falcon tubes. Formaldehyde reactivity was neutralized with 1% bovine serum albumin (which also blocks hydrophobic interactions in non-specific antibody binding), then platelets were incubated with primary antibodies and stain-labelled secondary antibodies. Different combinations of primary and secondary antibodies were used. The platelets were incubated with the primary antibodies diluted in PBS, 1% BSA, and 0.1% saponin. All monoclonal/polyclonal anti-platelet molecule antibodies, including anti-von Willebrand factor, tested so far were 1:100 except mouse anti-tubulin, which was 1:200 and rabbit serum anti-fibrocystin, which was 1:20. After washing, cover glasses were then incubated with secondary antibodies diluted in PBS + 1% BSA + 0.1% saponin at a dilution of about 1:200 or 300. After incubating 1 h in a wet chamber (protected from light), washed 3X PBS 0.1% saponin was then incubated in PBS (Falcon, Corning, VWR International, Dietikon, Switzerland) for 20 min. A drop of mounting medium was added to the slide and covered with the cover slip avoiding bubbles. Then, 10 μL were used for 12 mm-diameter cover slips. After drying (30 min–1 h), the cover-slip edge was sealed with nail varnish, allowed to dry, and checked for leaks. Slides were stored horizontally at 4 °C and protected from light in a dry polystyrol box.

Images were obtained using a Zeiss LSM910 fluorescence confocal microscope (Carl Zeiss AG, Oberkochen, Germany) (Figure 2).

### 3.13. 3D Structure Prediction via AlphaFold 

**Figure 7:**
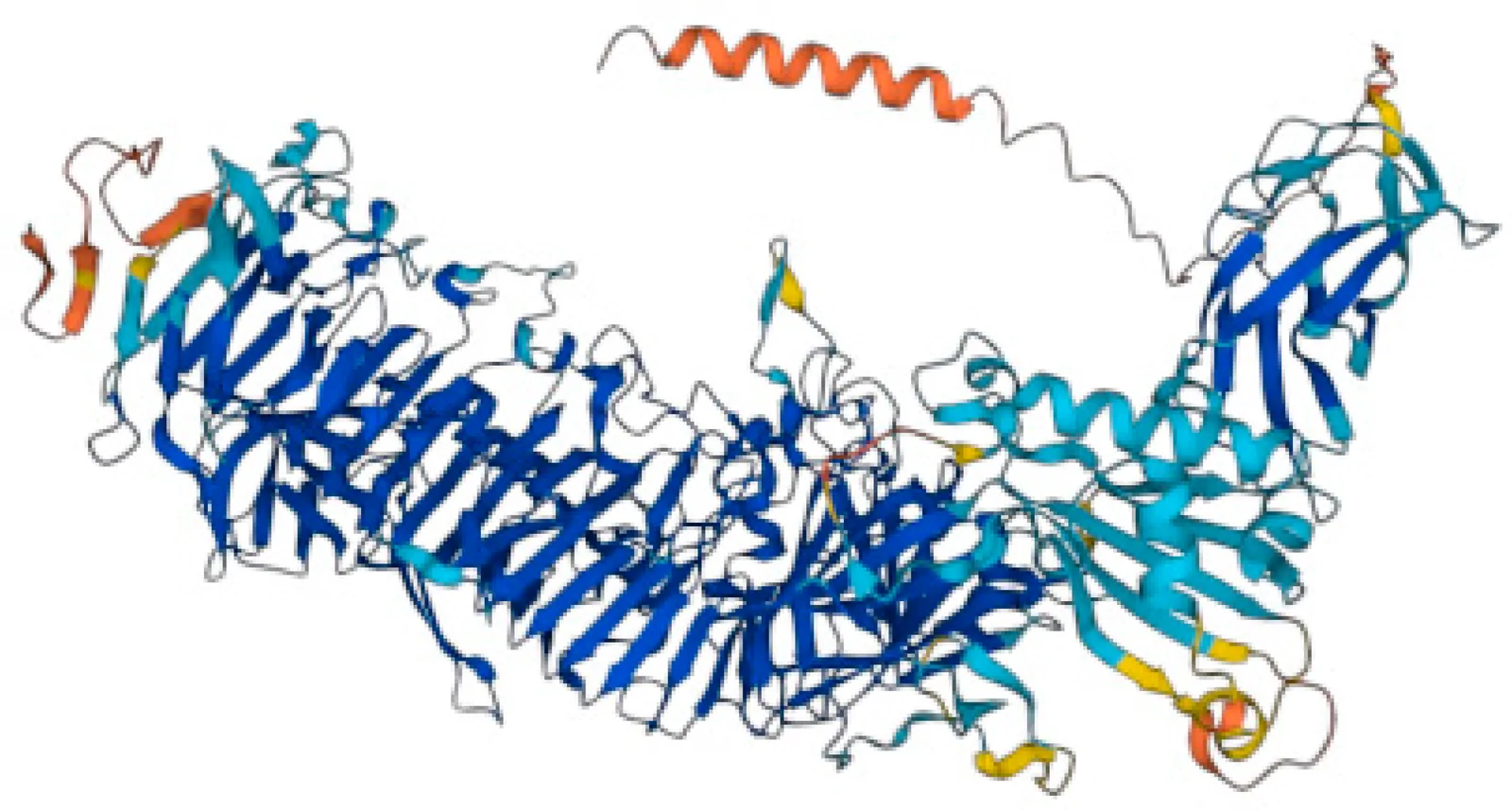
Structural model of fibrocystin L.

Structural model of fibrocystin L (Figure 7) as predicted by the AlphaFold structure prediction program [40,41]. Shown under the conditions of a CC-BY-4.0 license. Most of the structure in dark blue represents the immunoglobulin-like fold shared by plexins and transcription factors (TIG/IPT), which is also found in some structurally related molecules and has a high degree (above 96%) of accuracy in prediction. The α-helical segment in brown at the top is the C-terminal peptide ^1150^RMWLLEIFMAASTLNITLRSY^1171^ C-terminus, and methionine^1158^ was therefore predicted as the cleavage point and attachment site of the GPI anchor via GPI-transamidase using a Kohonen self-organizing map [30].

## Data Availability

The original contributions presented in this study are included in the article. Further inquiries can be directed to the corresponding author.
